# Quantized field with excitations of spacetime

**DOI:** 10.1038/s41598-025-16139-6

**Published:** 2025-08-22

**Authors:** Hou Yau

**Affiliations:** San Francisco State University, 1160 Holloway Avenue, 94132 San Francisco, CA USA

**Keywords:** Time and space symmetry, Excitation of spacetime, Oscillation in time, Uncertainty relation, Bosonic field, Schwarzschild field, Quantum physics, Physics, General relativity and gravity

## Abstract

We study a quantized field that can excite its underlying spacetime and has the properties of a bosonic field. A particle in this field is a harmonic oscillator in time, also known as a proper time oscillator, which is an excitation of spacetime. Time in this oscillator flows only forward but with varying rates. In separate analyses, by assuming the same proper time oscillator as a classical object that can remain stationary in space, we show that the spacetime outside is a Schwarzschild field. A classical proper time oscillator mimics the effects of a point mass in general relativity. As shown, a proper time oscillator has the properties of a quantum particle and can act as a gravitational source. Based on these results, if a real particle is an excitation of the corresponding quantum field and its underlying spacetime, the proper time oscillation will allow a real particle to interact directly with spacetime, generating a gravitational field.

## Introduction

According to the second law of thermodynamics, entropy tends to increase with time in an isolated system. All physical systems evolve over time, but no such translation over space necessarily exists. Despite these asymmetries, we are used to treating time and space on equal footing, the way they are treated in relativity. If a system demonstrates specific characteristics in space, we might also find systems (or in the same system) with similar characteristics in time. A simple example is the quantum mechanical plane wave, which exhibits periodicity in both time and space. Another example is time crystals^1,2^. In the theory of time crystals, a question is asked: If a regular crystal has broken spatial translational symmetry, could there be a time crystal with a time-periodic ground state that breaks the time translational symmetry? Even though several “no-go” theorems were presented^3–5^, which seem to forbid the creation of time crystals, the difficulties have been overcome, allowing time crystals to be experimentally realized^6–12^. Here, we have a similar yet different question. If a quantum harmonic oscillator oscillates in space, can there be an oscillator in time? To test if there is such a possibility, we investigate what properties the temporal oscillation can produce and compare them with those derived from quantum theory and general relativity.

In quantum field theory, particles are excitations of underlying quantum fields that permeate all of space. Here, we study a quantized real scalar field that can excite its underlying spacetime and with properties of a bosonic field. A particle in this field is a harmonic oscillator in time (also called proper time oscillator throughout this article); it has properties analogous to a quantum harmonic oscillator, albeit the oscillation is in time, not space.

A proper time oscillator is an excitation of spacetime. Its fluctuating time rate is a gravitational source and not a product of the time dilation due to relative motion or external gravitational field. Proper time oscillations can also be applied to a quantized field. As we will demonstrate, a proper time oscillator has the properties of a quantum particle and can generate a gravitational field. To clarify, when we speak of proper time oscillation, time flows only forward but with varying rates. Also, the fluctuations of spacetime we studied are much larger than the Planck scale, which differs from the fluctuations caused by spacetime foam. This paper is structured as follows:

“Proper time oscillation” section introduces the concepts of proper time oscillation. The basic properties of a proper time oscillator are defined.

“Quantum field with proper time oscillations” section illustrates the properties of a quantized real scalar field with spacetime excitations. The field has the same basic properties as a zero-spin bosonic field. The particles in the field are proper time oscillators^13^, which have a similar Hamiltonian equation, commutation relation, and uncertainty relation^14^ as a quantum harmonic oscillator. The proper time oscillator is an excitation of the underlying spacetime.

“Gravitational field with proper time oscillation as a source” section shows that a proper time oscillator, treated as a classical object stationary in space, can mimic the effects of a point mass in general relativity. As a part of the spacetime geometry, the proper time oscillator interacts with its surrounding spacetime; the resulting geometry is a Schwarzschild field^15,16^. Interestingly, the spacetime structure cloistered behind the singularity of this system is well-defined, which is the proper time oscillation.

“Forward in time” section examines the flow of time in a proper time oscillator. The internal time of a proper time oscillator can flow only forward in time. A proper time oscillator can only oscillate with a unique angular frequency $\omega _0$ and amplitude $\mathring{T}_0=1/\omega _0$. Even after considering the oscillation’s quantum effects, the internal time of a non-interacting proper time oscillator cannot jump to the future or past, except with the minimal oscillation displacement from the ’flowing’ coordinate time. An uncertainty relation between an oscillator’s internal time and internal time rate is discussed.

“Conservation of intrinsic energy and restoring action” section identifies a local spacetime mechanism responsible for the restoring action in a proper time oscillator. By examining the Hamiltonian equation, we show that the intrinsic mass-energy of a non-interacting proper time oscillator is conserved.

“Temporal and spatial oscillations symmetry” section clarifies that the symmetry considered in this paper is for harmonic oscillations and not coordinate time. The temporal displacement of a proper time oscillator can be reckoned as a self-adjoint operator^13^ without contradicting Pauli’s theorem^17,18^.

“Proper time oscillator vs. standard theories” section reviews the properties of a proper time oscillator against the quantum theory and general relativity. Within the limit that the oscillations are not large enough for detection, a proper time oscillator has the exact properties of a quantum particle.

“Magnitudes of the oscillations” section investigates the possibility that real particles are also proper time oscillators with their intrinsic quantum properties. If a real particle has proper time oscillation, it can allow a real particle to acquire its mass-energy and interact directly with spacetime, generating a gravitational field. Examination of the magnitude of these assumed oscillations for all known real particles reveals that they have yet to reach a level detectable by the current experiments. However, the oscillations can be magnified by projecting a real particle to high energy, making the oscillations easier to detect. Lastly, we examine the oscillations of neutrinos, which could be magnified to a macroscopic scale, providing a better chance of being detected in future experiments. The last section is reserved for conclusions and discussions.

## Proper time oscillation

What do we mean by oscillation in proper time? To illustrate the idea, let us consider an analogy with a particle traveling at an average velocity $\textbf{v}$. The particle also oscillates with an angular frequency $\omega$ and an amplitude $\mathring{\textbf{X}}$, i.e.,

1 $$\begin{aligned} \mathring{\textbf{x}}_f = \textbf{v}t-\mathring{\textbf{X}}\sin (\omega t), \end{aligned}$$

where $\mathring{\textbf{x}}_f$ is the position of the particle. To a stationary observer, the particle has varying velocities. Suppose the angular frequency $\omega$ is large, and the amplitude $\mathring{\textbf{X}}$ is small; the particle will appear to travel with a constant velocity if the instrument used by the observer is not sensitive enough to detect the slight variation of the oscillation. The properties of this model can be readily derived from classical mechanics. In this paper, we will investigate a similar model but replace a particle’s spatial motions with fluctuating time of the underlying spacetime.

Consider a coordinate system $(t,\textbf{x})$. The coordinate time *t* is measured by the clock of a stationary observer *O* at spatial infinity. For a Minkowski spacetime, a clock stationary anywhere in the coordinate system can be synchronized with the clock of *O*. Instead of being flat, let us assume time at the origin of spatial coordinates $\textbf{x}_0$ flows forward but oscillates with amplitude $\mathring{T}_0$ and angular frequency $\omega _0$. This fluctuating proper time of the underlying spacetime is,

2 $$\begin{aligned} \mathring{t}_f = t+\mathring{t}_{d} = t-\mathring{T}_0\sin (\omega _0 t), \end{aligned}$$

where

3 $$\begin{aligned} \mathring{t}_{d} = -\mathring{T}_0\sin (\omega _0 t). \end{aligned}$$

Unlike in a Minkowski spacetime, a clock at $\textbf{x}_0$ cannot be synchronized with the clock of *O* at spatial infinity.

As a part of the spacetime geometry, the fluctuating proper time at $\textbf{x}_0$ has geometrical properties that differ from those at spatial infinity, which is asymptotically flat with no oscillations. The difference in spacetime geometry at two spatially far apart locations implies that the spacetime between cannot be flat. The fluctuating proper time at $\textbf{x}_0$ can curve its surrounding spacetime and generate a gravitational field.

The fluctuation of spacetime at $\textbf{x}_0$ is a harmonic oscillator in time (or proper time oscillator). Here, we provide a classical description of the oscillator. Its quantum properties will be discussed in the next section. However, before we proceed, it is important to note that when we refer to proper time oscillation, time flows forward but with varying rates, as demonstrated in Eq. (2). Also, the fluctuations of spacetime we studied are much larger than the Planck scale, which differs from the fluctuations caused by spacetime foam.

The time read by a clock at the proper time oscillator will reflect the fluctuation in the underlying spacetime as shown in Eq. (2). The amplitude $\mathring{T}_0$ is analogous to the amplitude of a classical oscillator, except the oscillation is in time and not in space. The fluctuating time rate, defined as the derivative of the fluctuating time relative to the coordinate time, is

4 $$\begin{aligned} \mathring{R}_{t}=\frac{\partial \mathring{t}_f}{\partial t}=1-\mathring{T}_0 \omega _0\cos (\omega _0t), \end{aligned}$$

which has an average of 1. After averaging, the fluctuating time ‘flows’ at the same rate as the coordinate time.

On the other hand, the proper time oscillator is stationary at a specific coordinate $\textbf{x}=\textbf{x}_0$ of the spatial frame. An oscillator at rest has no spatial oscillation displacement, i.e., $\mathring{\textbf{x}}_d=0$. As shown, Eq. (2) is an analogy of Eq. (1), except a particle’s spatial motions are replaced by fluctuating time of the underlying spacetime. Note that the oscillation of a particle in space requires an external spring mechanism, as shown in the analogous example. In “Conservation of intrinsic energy and restoring action” section, we will identify a mechanism involving local spacetime responsible for the restoring action in a proper time oscillator. The total intrinsic mass-energy of a non-interacting oscillator is conserved. After a Lorentz boost, the total energy of the oscillations in time and space is also conserved.

The ’flowing’ coordinate time *t* labels the equilibrium position of the proper time oscillation. The temporal displacement $\mathring{t}_{d}$ from Eq. (3) is measured against this ‘equilibrium’. Suppose the clock of *O* at spatial infinity is not sensitive enough to pick up the proper time oscillator’s time fluctuation. To observer *O*, a particle at $\textbf{x}_0$ will appear to travel along a smooth geodesic as if there is no oscillation. In “Magnitudes of the oscillations” section, we will examine the magnitude of these oscillations. Assuming real particles are also proper time oscillators, our analyses reveal that their oscillations have yet to reach a level detectable by the current experiments.

The fluctuation of proper time in the underlying spacetime is not a product of the time dilation in a moving frame or gravitational field. (Similar idea of time fluctuation is also proposed in Ref.^19^.) The proper time oscillator is stationary, and no relative spatial motion causes the variation of time rate, i.e., $v=0$ in the time dilation equation. Also, the system we are considering is ’free’ with no force fields. No external gravitational field causes time dilation, i.e., gravitational potential $\phi =0$. A proper time oscillator is a gravitational source.

We can extend the properties of proper time oscillation to a plane wave as observed by *O*. Let us assume time everywhere in a plane wave ${\widehat{\zeta }_0}=(\zeta _{t0},\zeta _{\textbf{x 0}})$ flows forward but also oscillates in proper time; $\zeta _{t0}$ and $\zeta _{\textbf{x 0}}$ are temporal and spatial displacements in exponential form. This fluctuating proper time in the underlying spacetime is,

5 $$\begin{aligned} t_{f}(t,\textbf{x})=t+\text {Re}[\zeta _{t0}(t,\textbf{x})]=t-T_0\sin (\omega _0 t), \end{aligned}$$

where

6 $$\begin{aligned} \zeta _{t0}(t,\textbf{x})=-iT_0e^{-i\omega _0t}. \end{aligned}$$

The temporal displacement is,

7 $$\begin{aligned} t_{d}(t,\textbf{x})=\text {Re}[\zeta _{t0}(t,\textbf{x})]=-T_0\sin (\omega _0 t). \end{aligned}$$

The fluctuating proper time rate is,

8 $$\begin{aligned} R_t=\frac{\partial t_{f}(t,\textbf{x})}{\partial t}=1-\omega _0 T_0\cos (\omega _0 t), \end{aligned}$$

which has an average of 1. The average rate of fluctuating proper time is the same as the flow rate of the coordinate time. In addition, the underlying spacetime fluctuations have no spatial oscillations, i.e., $\textbf{x}_f(t,\textbf{x})=\textbf{x}$, ${\textbf{x}}_d(t,\textbf{x})=0$. and $\zeta _{\textbf{x 0}}(t,\textbf{x})=0$. As shown, plane wave $\widehat{\zeta }_0$ has properties analogous to the proper time oscillator, except we apply proper time oscillations to a plane wave.

In our current analysis, we will assume the gravitational effects generated by the fluctuating proper time are negligible. The background spacetime $(t, \textbf{x})$ can be treated as flat. Only the spacetime fluctuations $(t_f, \textbf{x}_f)$ underlying the plane wave are considered here. The gravitational effects of the fluctuating proper time will be discussed in “Gravitational field with proper time oscillation as a source” section.

The time read by a clock placed inside plane wave $\widehat{\zeta }_0$ will fluctuate as shown in Eq. (5). Suppose an event will happen at $(t,\textbf{x})$ if there is no fluctuating proper time. The same event will appear to happen at $(t_f,\textbf{x}_f=\textbf{x})$ inside plane wave $\widehat{\zeta }_0$ with proper time fluctuations. This property is analogous to a timelike interval, which varies depending on the strength of the gravitational field present. However, the fluctuating time in the underlying spacetime is not a product of an external gravitational field. The system we are considering is ’free’. Indeed, the fluctuating proper time can act as a gravitational source.

The displaced coordinates $(t_f,\textbf{x}_f)$ in frame *O* can be Lorentz transformed to the displaced coordinates $(t_f',\textbf{x}_f')$ as observed in another frame $O'$, i.e.,

9 $$\begin{aligned} & t'_f=t'+t'_d=t'+\text {Re}(\zeta _{t\textbf{k}})=t'+T_\textbf{k}\sin (\textbf{k}\cdot \textbf{x}'-\omega t'), \end{aligned}$$

10 $$\begin{aligned} & \textbf{x}'_f=\textbf{x}'+{\textbf{x}}'_d=\textbf{x}'+\text {Re}(\zeta _{\textbf{x}\textbf{k}})=\textbf{x}'+\textbf{X}_\textbf{k}\sin (\textbf{k}\cdot \textbf{x}'-\omega t'), \end{aligned}$$

where

11 $$\begin{aligned} & t'_d=\text {Re}(\zeta _{t\textbf{k}}), \quad {\textbf{x}}'_d= \text {Re}(\zeta _{\textbf{x}\textbf{k}}), \end{aligned}$$

12 $$\begin{aligned} & \zeta _{t\textbf{k}}=-iT_\textbf{k}e^{i(\textbf{k}\cdot \textbf{x}'-\omega t')}, \end{aligned}$$

13 $$\begin{aligned} & \zeta _{\textbf{x}\textbf{k}}=-i\textbf{X}_\textbf{k}e^{i(\textbf{k}\cdot \textbf{x}'-\omega t')}, \end{aligned}$$

14 $$\begin{aligned} & T_\textbf{k}=(\omega /\omega _0)T_{0}, \quad \textbf{X}_\textbf{k}=(\textbf{k}/\omega _0)T_{0}. \end{aligned}$$

We assume frame *O* travels at a velocity $\textbf{v}=\textbf{k}/\omega$ relative to frame $O'$ with $\gamma =1/\sqrt{1-\vert \textbf{v}\vert ^2}=\omega /\omega _0$. $(T_\textbf{k},\textbf{X}_\textbf{k})$ is a 4-amplitude Lorentz transformation of $(T_0,\textbf{0})$, i.e.,

15 $$\begin{aligned} \mid T_\textbf{k}\mid ^2=\mid T_{0} \mid ^2+\mid \textbf{X}_\textbf{k}\mid ^2, \end{aligned}$$

and the natural units $c=\hbar =1$ are adopted in this paper.

In the above analysis, we adopted the Lagrangian wave mechanics formulation. The temporal and spatial displacements $t'_d(t',\textbf{x}')$ and $\textbf{x}'_d(t',\textbf{x}')$ are measured against the undisturbed state $(t',\textbf{x}')$. In the Lagrangian formulation, $\textbf{x} '_d(t',\textbf{x}')$ tells us the spatial displacement from the undisturbed coordinate $\textbf{x}'$ at time $t'$. Similarly, $t'_d (t',\textbf{x}')$ is the difference between the fluctuating time and the coordinate time $t'$. A clock originally at $\textbf{x}'$ and $t'=0$ will be displaced to $\textbf{x}'_f=\textbf{x}'+\textbf{x}'_d$, and measures a fluctuate time $t'_f=t'+t'_d$.

As observed in frame $O'$, the spacetime underlying plane wave $\widehat{\zeta }_\textbf{k}=(\zeta _{t\textbf{k}},\zeta _{\textbf{x}\textbf{k}})$ oscillates in time and space. Suppose an event will happen at $(t',\textbf{x}')$ if there are no fluctuations in spacetime. The same event will appear to happen at $(t_f',\textbf{x}_f')$ inside plane wave $\widehat{\zeta }_\textbf{k}$ with spacetime fluctuations. The fluctuating time $t_f'$ is measured by a clock originally placed at $\textbf{x}'$ and $t'=0$. As time evolves, the clock oscillates relative to the equilibrium at $\textbf{x}'$ in the spatial frame. Because of the fluctuations in the underlying spacetime, a particle traveling through this plane wave $\widehat{\zeta }_\textbf{k}$ will have oscillations along its path.

Writing as a column vector,

16 $$\begin{aligned} \left[ \begin{array}{ c } \zeta _{t\textbf{k}} \\ \zeta _{\textbf{x}\textbf{k}} \end{array} \right] = -i \left[ \begin{array}{ c } T_\textbf{k} \\ \textbf{X}_\textbf{k} \end{array} \right] e^{i(\textbf{k}\cdot \textbf{x}'-\omega t')}, \end{aligned}$$

this Lorentz covariant plane wave describes a wave with an angular wavenumber $\textbf{k}$ that has spacetime oscillations. By the superposition theory, a wave packet can be created by superposing Lorentz covariant plane waves with different $\textbf{k}$. As discussed previously, we assume the gravitational effects of the oscillations are negligible, and the background spacetime $(t', \textbf{x}')$ is considered flat when applying the Lorentz covariant plane wave in our current application.

## Quantum field with proper time oscillations

In the previous section, we defined proper time oscillation. Our next task is to investigate what properties the temporal oscillation can produce and compare them with those of a quantum field.

### Plane wave with oscillations in time and space

Let us define a plane wave,

17 $$\begin{aligned} \zeta _\textbf{k}=\frac{T_{0\textbf{k}}}{\omega _0}e^{i(\textbf{k}\cdot \textbf{x}-\omega t)}. \end{aligned}$$

Here, we use $T_{0\textbf{k}}$ to distinguish the proper time wave amplitudes for plane waves with different angular wavenumber $\textbf{k}$. From Eqs. (12) and (13), the temporal and spatial displacements in exponential form can be written in terms of $\zeta _\textbf{k}$, i.e.

18 $$\begin{aligned} \zeta _{t\textbf{k}}= & \partial _0\zeta _\textbf{k}=-iT_\textbf{k}e^{i(\textbf{k}\cdot \textbf{x}-\omega t)}, \end{aligned}$$

19 $$\begin{aligned} \zeta _{\textbf{x}\textbf{k}}= & -\mathbf {\nabla }\zeta _\textbf{k}=-i\textbf{X}_\textbf{k}e^{i(\textbf{k}\cdot \textbf{x}-\omega t)}. \end{aligned}$$

Therefore, $\zeta _{\textbf{k}}$ is a plane wave with both temporal and spatial oscillations. To obtain the oscillations, we simply take the derivatives of $\zeta _{\textbf{k}}$ as shown in Eqs. (18) and (19). (For convenience purposes, we have dropped the prime symbol from the coordinate $(t',\textbf{x}')$ in the rest of the paper.)

The plane wave $\zeta _{\textbf{k}}$ and its conjugate $\zeta _{\textbf{k}}^*$ satisfy the Klein Gordon equation:

20 $$\begin{aligned} & \partial _u\partial ^u\zeta _{\textbf{k}} +\omega _0^2\zeta _{\textbf{k}}=0, \end{aligned}$$

21 $$\begin{aligned} & \partial _u\partial ^u\zeta _{\textbf{k}}^*+\omega _0^2\zeta _{\textbf{k}}^*=0. \end{aligned}$$

The Lagrangian density and Hamiltonian density of $\zeta _{\textbf{k}}$ defined within a cubic box of volume *V* are,

22 $$\begin{aligned} & \mathcal {L}_{\textbf{k}}=\left( \frac{K}{2V} \right) [(\partial ^u\zeta _{\textbf{k}}^*)(\partial _u\zeta _{\textbf{k}})-\omega _0^2\zeta _{\textbf{k}}^*\zeta _{\textbf{k}}], \end{aligned}$$

23 $$\begin{aligned} & \mathcal {H}_{\textbf{k}}=\left( \frac{K}{2V} \right) [(\partial _0\zeta _{\textbf{k}}^*)(\partial _0\zeta _{\textbf{k}})+(\mathbf {\nabla }\zeta _{\textbf{k}}^*)\cdot (\mathbf {\nabla }\zeta _{\textbf{k}})+\omega _0^2\zeta _{\textbf{k}}^*\zeta _{\textbf{k}}], \end{aligned}$$

where *K* is a constant of the system we are investigating, periodic boundary conditions are imposed on the box walls. The factor of 1/2 is inserted for ease of demonstration.

Substitute $\zeta _{\textbf{k}}$ from Eq. (17) into Eq. (23), the Hamiltonian density of a plane wave is,

24 $$\begin{aligned} \mathcal {H}_{\textbf{k}}=\mathcal {H}_1+\mathcal {H}_2+\mathcal {H}_3, \end{aligned}$$

where

25 $$\begin{aligned} \mathcal {H}_1= & \left( \frac{K}{2V} \right) T_{\textbf{k}}^*T_{\textbf{k}}, \end{aligned}$$

26 $$\begin{aligned} \mathcal {H}_2= & \left( \frac{K}{2V} \right) {\textbf{X}}_{\textbf{k}}^*\cdot {\textbf{X}}_{\textbf{k}}, \end{aligned}$$

27 $$\begin{aligned} \mathcal {H}_3= & \left( \frac{K}{2V} \right) T_{0\textbf{k}}^*T_{0\textbf{k}}. \end{aligned}$$

As we shall note, $\mathcal {H}_1$, $\mathcal {H}_2$ and $\mathcal {H}_3$ are the Hamiltonian densities associated with oscillations in time, space, and proper time. Within the non-relativistic limit, $\mathcal {H}_2$ has the form of the Hamiltonian density for a classical harmonic system. The Hamiltonian densities of the temporal oscillations, $\mathcal {H}_1$ and $\mathcal {H}_3$, have similar equations as $\mathcal {H}_2$, except the spatial amplitude is replaced by the temporal amplitudes.

In relativity, a gravitational wave is mathematically described as a rank-2 tensor, reflecting how it stretches and squeezes space as it passes through. Time dilation caused by gravity and motion changes the rate of time; the stronger the gravity or the faster the motion, the slower time passes relative to a stationary observer with weaker gravity or slower speed. However, as discussed in “Proper time oscillation” section, a ’stationary’ and ’free’ proper time oscillator has a fluctuating time rate that is not the product of an external gravitational field or motion. The Lorentz covariant plane wave defined in Eq. (16) is a rank-1 tensor and differs from the rank-2 tensor of a gravitational wave. On that account, what physical systems can cause their underlying spacetime to fluctuate?

A classical proper time oscillator has the gravitational effects of a point mass in relativity. The Schwarzschild field of a classical proper time oscillator will be discussed in “Gravitational field with proper time oscillation as a source” section. That being the case, a black hole can curve the surrounding spacetime if its underlying time fluctuates. However, due to the extreme nature of the black hole’s singularity, where gravity is theoretically infinite, we cannot directly observe or measure what occurs at that point. Whether a black hole has proper time oscillations requires a better understanding of the quantum gravity theory.

We can also search for systems at the quantum level. Quantum mechanics features unique properties like quantum tunneling, quantum interference, and wave-particle duality that are not observed in the macroscopic realm. In quantum field theory, particles are excitations of their corresponding underlying quantum fields. If spacetime can be excited with fluctuating time, as shown, a quantized field could exist that can excite its underlying spacetime. The quanta created will be proper time oscillators. This field is the system we will investigate next.

To relate the spacetime oscillations of plane wave $\zeta _{\textbf{k}}$ with a quantized field, we make an ansatz

28 $$\begin{aligned} K=m\omega _0^2, \end{aligned}$$

where *m* is the mass of particles created in the system; $\omega _0$ is the de Broglie’s angular frequency of the particle’s rest mass-energy^20^. Note that the de Broglie’s angular frequency of the rest mass-energy is an intrinsic property and a fundamental characteristic inherent to a particle. It is reasonable to assume that the proper time oscillation, another intrinsic property, would oscillate at the same frequency. Also, $K=m\omega _0^2$ has the familiar form of the spring constant for a classical simple harmonic oscillator.

### Quantized real scalar field

For our current analysis, it is more convenient to use a normalized plane wave $\tilde{\zeta }_\textbf{k}$ derived from $\zeta _{\textbf{k}}$, i.e.,

29 $$\begin{aligned} \tilde{\zeta }_\textbf{k}=\sqrt{\frac{\omega _0}{\omega }}\zeta _\textbf{k}=\frac{T_{0\textbf{k}}}{\sqrt{\omega _0\omega }}e^{i(\textbf{k}\cdot \textbf{x}-\omega t)}, \end{aligned}$$

and $\sqrt{\omega _0/\omega }$ is a normalization factor. From Eqs. (12), (13) and (14), the spacetime oscillations’ amplitudes underlying this plane wave are,

30 $$\begin{aligned} & {\tilde{T}}_{\textbf{k}}=\sqrt{\frac{\omega _0}{\omega }} T_{\textbf{k}}=\sqrt{\frac{\omega }{\omega _0}} T_{0\textbf{k}}, \end{aligned}$$

31 $$\begin{aligned} & \tilde{\textbf{X}}_{\textbf{k}}=\sqrt{\frac{\omega _0}{\omega }}{\textbf{X}}_{\textbf{k}}=\frac{\textbf{k}}{\sqrt{\omega _0\omega }}T_{0\textbf{k}}. \end{aligned}$$

Here, we can construct a real scalar field by superposition of the normalized plane waves ${\tilde{\zeta }}_{\textbf{k}}$ and their conjugates ${\tilde{\zeta }}^*_{\textbf{k}}$, i.e.,

32 $$\begin{aligned} \zeta ({x}) = \sum \limits _{\textbf{k}} \frac{\tilde{\zeta }_{\textbf{k}} + \tilde{\zeta }^*_{\textbf{k}}}{\sqrt{2}}= \sum \limits _{\textbf{k}}(2\omega \omega _0)^{-1/2} (T_{0\textbf{k}} e^{-i{k} {x}} + T^*_{0\textbf{k}} e^{i{k}{x}}). \end{aligned}$$

The real scalar field $\zeta ({x})$ satisfies Klein–Gordon equation. Based on the Lagrangian density from Eq. (22), the conjugate momenta of $\zeta ({x})$ is,

33 $$\begin{aligned} \eta ({x})=\frac{\partial \mathcal {L}}{\partial [\partial _0\zeta ({x})]}=\frac{-i}{V}\sqrt{\frac{\omega _0^5 \omega }{2}} \sum \limits _{\textbf{k}} [T_{0\textbf{k}} e^{-i{k}{x}}-T_{0\textbf{k}}^*e^{i{k} {x}}]. \end{aligned}$$

The system we are considering is a cube with volume *V*, and periodic boundary conditions are imposed on the box walls.

To look for hints on what quantum systems can cause their underlying spacetime to fluctuate, we will try quantizing the real scalar field $\zeta (x)$ and analyze its properties. Following the concepts developed in quantum theory, we can transform a classical field into a quantized field via canonical quantization. In other words, $\zeta ({x})$, $\eta ({x})$, $T_{0\textbf{k}}$ and $T_{0\textbf{k}}^*$ can be promoted to quantum operators with suitable commutation relations, allowing for the description of the field in terms of particles or excitations. The quantization process of $\zeta ({x})$ is similar to the bosonic field $\varphi ({x})$, as we can see that they are related by

34 $$\begin{aligned} \varphi ({x})=\zeta ({x})\sqrt{\frac{\omega _0^3}{V}}= \sum \limits _{\textbf{k}}(2 \omega V)^{-1/2} (a_\textbf{k} e^{-i{k} {x}} + a^\dagger _\textbf{k} e^{i{k} {x}}), \end{aligned}$$

where

35 $$\begin{aligned} a_\textbf{k}=\omega _0 T_{0\textbf{k}}, \quad a^\dagger _\textbf{k}=\omega _0 T^\dagger _{0\textbf{k}}, \end{aligned}$$

are the annihilation and creation operators.

As a quantized field, $\zeta ({x})$ and $\eta ({x})$ satisfy the equal-time commutation relations,

36 $$\begin{aligned} & {[}\zeta (t,\textbf{x}),\eta (t,\textbf{x}')]=i \delta (\textbf{x}-\textbf{x}'), \end{aligned}$$

37 $$\begin{aligned} & {[}\zeta (t,\textbf{x}),\zeta (t,\textbf{x}')]=[\eta (t,\textbf{x}),\eta (t,\textbf{x}')]=0. \end{aligned}$$

The operators $T_{0\textbf{k}}$ and $T^\dagger _{0\textbf{k}}$ satisfy the commutation relations:

38 $$\begin{aligned} {[}T_{0\textbf{k}},T^\dagger _{0\mathbf {k'}}]= & \frac{[a_{\textbf{k}},a^\dagger _{\mathbf {k'}}]}{\omega _0^2}=\frac{\delta _{\textbf{k}\mathbf {k'}}}{\omega _0^2}, \end{aligned}$$

39 $$\begin{aligned} {[}T_{0\textbf{k}},T_{0\mathbf {k'}}]= & [T^\dagger _{0\textbf{k}},T^\dagger _{0\mathbf {k'}}]=\frac{[a_{\textbf{k}},a_{\mathbf {k'}}]}{\omega _0^2}=\frac{[a^\dagger _{\textbf{k}},a^\dagger _{\mathbf {k'}}]}{\omega _0^2}=0. \end{aligned}$$

Substituite $\zeta ({x})$ into Eq. (23), the Hamiltonian of the field inside volume *V* is,

40 $$\begin{aligned} H= \sum \limits _{\textbf{k}} \omega (\omega _0^2 T^\dagger _{0\textbf{k}} T_{0\textbf{k}}+ \frac{1}{2})= \sum \limits _{\textbf{k}} \omega (a^\dagger _{\textbf{k}} a_{\textbf{k}} + \frac{1}{2}), \end{aligned}$$

which is the same Hamiltonian obtained in a bosonic field. Based on Eqs. (12) and (13), the temporal and spatial oscillation displacements in the underlying spacetime are,

41 $$\begin{aligned} t_d(x)= & \zeta _{t}(x)=\partial _0\zeta (x)=\sum \limits _{\textbf{k}}\frac{-i}{\sqrt{2}}({\tilde{T}}_{\textbf{k}} e^{-i{k} {x}} - {\tilde{T}}^\dagger _{\textbf{k}} e^{i{k}{x}}), \end{aligned}$$

42 $$\begin{aligned} \textbf{x}_d(x)= & \zeta _{\textbf{x}}(x)=-\mathbf {\nabla }\zeta (x)= \sum \limits _{\textbf{k}}\frac{-i}{\sqrt{2}}(\tilde{\textbf{X}}_{\textbf{k}} e^{-i{k} {x}} - \tilde{\textbf{X}}^\dagger _{\textbf{k}} e^{i{k}{x}}), \end{aligned}$$

where ${\tilde{T}}_{\textbf{k}}$ and $\tilde{\textbf{X}}_{\textbf{k}}$ are defined in Eqs. (30) and (31). Note that $\zeta _{t}(x)$ and $\zeta _{\textbf{x}}(x)$ are real in a real scalar field.

As demonstrated, the quantized real scalar field $\zeta (x)$ has the structures of a bosonic field. The properties of $\zeta ({x})$ can be obtained from the standard quantum theory by replacing the annihilation and creation operators with the proper time wave amplitudes. For example, the particle number operator is,

43 $$\begin{aligned} N=\sum \limits _{\textbf{k}}N_{\textbf{k}}, \end{aligned}$$

where

44 $$\begin{aligned} N_{\textbf{k}}=a^\dagger _{\textbf{k}} a_{\textbf{k}}=\omega _0^2 T^\dagger _{0\textbf{k}} T_{0\textbf{k}}. \end{aligned}$$

The spectrum of a particle number operator $N_{\textbf{k}}$ consists of all non-negative integers (0, 1, 2, 3, ...).

### Excitation of spacetime

The particles created in the real scalar field $\zeta (x)$ are proper time oscillators. They are excitations of the underlying spacetime that permeate all of space; essentially, ripples in spacetime carry energy. As a lump of energy, it is point-like and behaves as a particle. As an oscillator, it is a fluctuation of the underlying spacetime, which is a gravitational source. In our analysis, we will identify a proper time oscillator as a particle or oscillator, depending on its functions and applications.

For the single-particle states,

45 $$\begin{aligned} \vert \textbf{k}\rangle = a^\dagger _\textbf{k} \vert 0 \rangle , \end{aligned}$$

the square of their proper time amplitude is,

46 $$\begin{aligned} \mathring{T}_0^2=\langle \textbf{k}\vert T^\dagger _{0\textbf{k}} T_{0\textbf{k}}\vert \textbf{k}\rangle =\langle \textbf{k}\vert \frac{N_{\textbf{k}}}{\omega _0^2}\vert \textbf{k}\rangle =\frac{1}{\omega _0^2}. \end{aligned}$$

Therefore, oscillators of different momenta in $\zeta (x)$ oscillate with the same angular frequency $\omega _0$ and proper time amplitude in their rest frames,

47 $$\begin{aligned} \mathring{T}_0=1/\omega _0. \end{aligned}$$

This unique amplitude depends only on the oscillator’s mass-energy angular frequency. As a particle, each oscillator has an intrinsic mass-energy $\omega _0$ generated from the spacetime oscillation.

From Eqs. (41) and (42), the square of the temporal and spatial amplitudes for an oscillator with momentum $\textbf{k}$ are,

48 $$\begin{aligned} & \mathring{T}_\textbf{k}^2=\langle \textbf{k}\vert \zeta _t(x)^2\vert \textbf{k}\rangle =\frac{\omega }{\omega _0^3}, \end{aligned}$$

49 $$\begin{aligned} & \vert \mathring{\textbf{X}}_\textbf{k} \vert ^2=\langle \textbf{k}\vert \zeta _{\textbf{x}}(x)^2\vert \textbf{k}\rangle =\frac{\textbf{k}\cdot \textbf{k}}{\omega _0^3 \omega }. \end{aligned}$$

Normal ordering of $T^\dagger _{0\textbf{k}}$ and $T_{0\textbf{k}}$ has been taken. Therefore, the amplitudes of the spacetime oscillations are,

50 $$\begin{aligned} \mathring{T}_\textbf{k}=\sqrt{\frac{\omega }{\omega _0^3}},\quad \mathring{\textbf{X}}_\textbf{k}=\frac{\textbf{k}}{\sqrt{\omega _0^3\omega }}. \end{aligned}$$

The spatial oscillation is in the longitudinal direction of the propagation. As a particle, each oscillator carries an energy $\omega$. Following the same arguments, a *n*-particle state,

51 $$\begin{aligned} \vert \textbf{k}_1,......,\textbf{k}_n \rangle = a^\dagger _{\textbf{k}_1}......a^\dagger _{\textbf{k}_n} \vert 0 \rangle , \end{aligned}$$

is consisted of *n* proper time oscillators, each oscillates with the same unique proper time amplitude $\mathring{T}_0$ in their rest frames.

The quantized real scalar field $\zeta (x)$ with spacetime excitations is an infinite array of oscillators. It can be considered a matter field and has the basic properties of a bosonic field^13,14^. The difference is that the former has oscillations in time and space. Under the condition that the oscillations in time and space are small and not detectable, the quantized field $\zeta (x)$ can be treated as a bosonic field $\varphi (x)$ as expressed in Eq. (34). The bosonic field $\varphi (x)$ and its momenta conjugate $\pi (x)$ satisfy the equal-time commutation relations in the standard quantum theory. The Hamiltonian and the particle number operators are defined in Eqs. (40) and (44) with the creation and annihilation operators $a^\dagger _{\textbf{k}}$ and $a_{\textbf{k}}$. As will be discussed in “Magnitudes of the oscillations” section, if a real particle is an excitation of the corresponding quantum field and its underlying spacetime, the proper time oscillation will allow a real particle to acquire its mass-energy and interact directly with spacetime, generating a gravitational field.

From Eq. (35), the operators $T^\dagger _{0\textbf{k}}$ and $T_{0\textbf{k}}$ respectively create and annihilate a proper time oscillator, representing excitations of the underlying spacetime. These quanta exhibit oscillations in both time and space, characterized by the amplitudes $\mathring{T}_\textbf{k}$ and $\mathring{\textbf{X}}_\textbf{k}$, respectively. These oscillatory amplitudes are physically measurable quantities and reflect the inherently dynamic nature of spacetime in our framework. As we will discuss in “Magnitudes of the oscillations” section, temporal oscillations can manifest in the decay rates of unstable particles, while spatial oscillations influence a particle’s time of arrival. Together, these effects offer potential observational signatures of the proper time oscillator.

### Quantum harmonic oscillator in time

To further understand the properties of a proper time oscillator, let us consider a quantized real scalar field that has oscillations in proper time only, i.e.,

52 $$\begin{aligned} \zeta _0'(x) = \frac{1}{\sqrt{2}}(\tilde{\zeta }_0 + \tilde{\zeta }_0^\dagger )=\frac{1}{\sqrt{2}\omega _0} (T_0 e^{-i{\omega _0} t}+T_0^\dagger e^{i{\omega _0}t}). \end{aligned}$$

Applying Eq. (18), the temporal displacement $t_{d}$ and the temporal displacement rate $u_{d}$ are,

53 $$\begin{aligned} t_d= & \zeta _{t0}'=\partial _0\zeta _0'=\frac{-i}{\sqrt{2}} (T_0 e^{-i{\omega _0} t}-T_0^\dagger e^{i{\omega _0}t})\nonumber \\= & \frac{-i}{\sqrt{2}\omega _0} (a_0 e^{-i{\omega _0} t}-a_0^\dagger e^{i{\omega _0}t}), \end{aligned}$$

54 $$\begin{aligned} u_d= & \partial _0t_d=\frac{-\omega _0}{\sqrt{2}} (T_0 e^{-i{\omega _0} t}+T_0^\dagger e^{i{\omega _0}t})\nonumber \\= & \frac{-1}{\sqrt{2}} (a_0 e^{-i{\omega _0} t}+a_0^\dagger e^{i{\omega _0}t}). \end{aligned}$$

An oscillator created in this field oscillates with a unique amplitude $\mathring{T}_0 =1/\omega _0$ as demonstrated in Eq. (47); it is a proper time oscillator.

A proper time oscillator in $\zeta _0'(x)$ has no motion or oscillation in space. Therefore, the energy generated by the proper time oscillations shall belong to some intrinsic energy of the system. However, the system we are considering has no non-gravitational properties. The only intrinsic energy generated in this field is the mass-energy of the proper time oscillators.

From Eqs. (23) and (28), the Hamiltonian of the system is,

55 $$\begin{aligned} H_0=m \left( a_0^\dagger a_0+\frac{1}{2}\right) =m \left( \omega _0^2 T^\dagger _{0} T_{0}+ \frac{1}{2}\right) , \end{aligned}$$

which can be written in terms of $t_d$ and $u_d$, i.e.,

56 $$\begin{aligned} H_0=\frac{m\omega _0^2 {t_d}^2}{2} + \frac{{P_t}^2}{2m}, \end{aligned}$$

where

57 $$\begin{aligned} P_t=mu_d. \end{aligned}$$

This result is analogous to the Hamiltonian of a quantum harmonic oscillator, except the spatial oscillation is replaced by the temporal oscillation. The two terms on RHS of Eq. (56) are the semblance of a typical harmonic oscillator’s ‘potential’ and ‘kinetic’ energy. For a one-particle state, the mass-energy of an oscillator is generated by its oscillation in time with angular frequency $\omega _0$ and amplitude $\mathring{T}_0$.

The operator $P_t$ takes the role of ‘momentum’. The temporal displacement $t_d$ and the ‘temporal momentum’ $P_t$ are analogies of a quantum harmonic oscillator’s position and momentum operators. However, we stress that this $P_t$ is not the 0-component of the 4-momentum; it differs from the field’s energy.

Using the standard properties of the creation and annihilation operators, the temporal displacement $t_d$ and temporal momentum $P_t$ satisfy a commutation relation,

58 $$\begin{aligned} {[}t_d,P_t]=i. \end{aligned}$$

The result is analogous to the commutation relation between a quantum harmonic oscillator’s spatial displacement and momentum. Both $t_d$ and $P_t$ are self-adjoint operators. (Note that $t_d$ forms a conjugate pair with $P_t$ and not with the Hamiltonian $H_0$. As we will explain in “Temporal and spatial oscillations symmetry” section, the treatment of the temporal displacement $t_d$ as a self-adjoint operator^13^ does not contradict Pauli’s theorem^17,18^.)

The temporal displacement $t_d$ and the temporal momentum $P_t$ satisfy an uncertainty relation^14^,

59 $$\begin{aligned} \Delta t_d\Delta P_t=n+{\frac{1}{2}}\ge \frac{1}{2}, \end{aligned}$$

which is obtained from the temporal displacement and temporal momentum variances,

60 $$\begin{aligned} \Delta t_d= & \sqrt{\langle ( t_d)^2 \rangle - \langle t_d\rangle ^2}=\frac{1}{\omega _0} \sqrt{\left( n+\frac{1}{2} \right) }, \end{aligned}$$

61 $$\begin{aligned} \Delta P_t= & \sqrt{\langle ( P_t)^2 \rangle - \langle P_t\rangle ^2}=\omega _0 \sqrt{\left( n+\frac{1}{2} \right) }. \end{aligned}$$

Here, we used the de Broglie’s mass-energy $m=\omega _0$. Compare Eq. (59) with $\Delta x\Delta p\ge \frac{1}{2}$, the proper time oscillator satisfies an uncertainty relation that is similar to the one obtained for a quantum harmonic oscillator. The temporal uncertainty relation will be further discussed in “Forward in time” section.

As demonstrated, a field with spacetime excitations has the properties of a quantum field. The quanta observed in the system are proper time oscillators (or harmonic oscillators in time). Their properties, including Hamiltonian, commutation relation, and uncertainty relation, are similar to those of a quantum harmonic oscillator. In “Magnitudes of the oscillations” section, we will investigate another possibility that real particles could also have proper time oscillations and examine the magnitude of those oscillations. Conservation of energy and restoring action in a proper time oscillator will be discussed in “Conservation of intrinsic energy and restoring action” section.

## Gravitational field with proper time oscillation as a source

In the previous section, we demonstrate that a particle in a quantized real scalar field $\zeta (x)$ is an excitation of the underlying spacetime with oscillation in proper time. It is a proper time oscillator but it is not just ’sitting’ on a Minkowski spacetime. The proper time oscillation is a part of the spacetime geometry. Here, we investigate the gravitational effects of the proper time oscillator.

In the following analysis, we will neglect all quantum effects and treat the proper time oscillator as classical. Eq. (2) with $\mathring{T}_0=1/\omega _0$ describes the fluctuating time of the oscillator,

62 $$\begin{aligned} \mathring{t}_{f}=t+\mathring{t}_{d}=t-\frac{\sin (\omega _0 t)}{\omega _0}, \end{aligned}$$

which we will idealize here to be stationary at the spatial origin $\textbf{x}_0$ of a coordinate system $(t,\textbf{x})$. Apart from this proper time oscillator, there is no oscillation in time elsewhere. Treating the proper time oscillator as a stationary classical object, it shall have effects equivalent to those for a point mass at rest in general relativity, which we want to demonstrate in this section. Note that there are no general preferred coordinate systems in general relativity. The coordinate system used here will be later identified as Schwarzschild for convenience in the analysis.

### Fictitious oscillations

The proper time oscillation at $\textbf{x}_0$ is a pulse that can be decomposed into a series of plane waves. For a relativistic theory, we shall utilize Lorentz covariant plane waves for the decomposition, i.e.,

63 $$\begin{aligned} \left[ \begin{array}{ c } \bar{\xi }_{t\textbf{k}} \\ \bar{\xi }_{\textbf{x}\textbf{k}} \end{array} \right] = -i \left[ \begin{array}{ c } \bar{T}_\textbf{k} \\ \bar{\textbf{X}}_\textbf{k} \end{array} \right] e^{i(\textbf{k}\cdot \textbf{x}-\omega t)}. \end{aligned}$$

In “Proper time oscillation” and “Quantum field with proper time oscillations” sections, we applied a similar plane wave when we studied the real scalar field $\zeta (x)$ as a matter field. However, gravitational effects are neglected, and the background spacetime is considered flat. Only the spacetime oscillations underlying the field have been considered. For our current applications, instead of applying Lorentz covariant plane waves to describe only the spacetime oscillations underlying matter, we will use them to characterize the fluctuations in spacetime geometry outside the proper time oscillator. Top bars are added to the plane waves and amplitude symbols to distinguish our current application from the previous one for a matter field.

We can apply $\bar{\xi }_{t\textbf{k}}$ from Eq. (63) to carry out the decomposition for the proper time oscillation at $\textbf{x}_0$. However, $\bar{\xi }_{t\textbf{k}}$ is only the 0-component of a Lorentz covariant plane wave; the spatial component $\bar{\xi }_{\textbf{x}\textbf{k}}$ cannot be neglected. Thus, if we superpose the plane waves $\bar{\xi }_{t\textbf{k}}$ to obtain the proper time oscillation at $\textbf{x}_0$, there will be spatial oscillations associated with the superposition of $\bar{\xi }_{\textbf{x}\textbf{k}}$. These spatial oscillations are essential in our relativistic theory.

In spherical coordinates, the proper time oscillation and the radial oscillations revealed after the superposition are summarized as follows:

At $$r=0$$,

64 $$\begin{aligned} & \bar{t}_f(t,0)=t-\frac{\sin (\omega _0 t)}{\omega _0}, \end{aligned}$$

65 $$\begin{aligned} & \bar{r}_f(t,0)=0. \end{aligned}$$

At $$r=\epsilon /2 \rightarrow 0$$,

66 $$\begin{aligned} & \bar{t}_f(t,\epsilon /2)=t, \end{aligned}$$

67 $$\begin{aligned} & \bar{r}_f(t,\epsilon /2)=\epsilon /2+\Re _\infty \cos (\omega _0 t), \end{aligned}$$

where $\Re _\infty$ is the amplitude of radial oscillations, and its magnitude is approaching infinity ($\Re _\infty \rightarrow \infty$). Based on our convention adopted, $\bar{t}_f(t,r)$ and $\bar{r}_f(t,r)$ are the time and spatial position observed in the spacetime geometry displaced from the equilibrium state (*t*, *r*). Outside the sphere with $r=\epsilon /2$, the spacetime is a vacuum with no oscillations. (Details for the decomposition analysis can be found in ref.^16^)

Equation (64) describes the proper time oscillation in the underlying spacetime geometry caused by the field excitation. The proper time oscillation is stationary at the spatial origin, as demonstrated in Eq. (65). The radial oscillations from Eqs. (67) are the results of superposing the spatial component of the Lorentz covariant plane waves. These radial oscillations oscillate about a thin shell $\Sigma _0$ with infinitesimal radius ($r=\epsilon /2 \rightarrow 0$) centered at the origin. As we shall note, the amplitude of the radial oscillation has a magnitude approaching infinity ($\Re _\infty \rightarrow \infty$), which implies the instantaneous radial velocity is also approaching infinity. This result will violate the principles of relativity if the oscillations involve motions of matter. In fact, the only matter presents in this system is the particle at $\textbf{x}_0$. The outside spacetime is a vacuum. Therefore, the radial oscillations are not motions that carry matter through space. Instead, the radial oscillation is a spacetime geometrical effect acting on an observer stationary on the thin shell $\Sigma _0$.

In Minkowski spacetime, a clock stationary anywhere in the coordinate system can be synchronized with the clock of a stationary observer *O* at spatial infinity. However, this is not the case for an observer $\breve{O}$ stationary on the thin shell $\Sigma _0$. As shown in Eq. (66), the clock of *O* is synchronized with the clock of a ’fictitious’ observer $\underline{O}$ that follows the radial oscillation defined in Eq. (67). The oscillating observer $\underline{O}$ measures the coordinate time as an inertial observer. This result demonstrates something unique about the spacetime geometry outside the proper time oscillation. The system has inertial frames oscillating relative to a stationary observer in the coordinate system. We will call these oscillating inertial frames ’fictitious inertial frames’.

An observer $\breve{O}$ placed on the thin shell will oscillate relative to $\underline{O}$. Since the clocks of *O* and $\underline{O}$ are synchronized, the clocks of $\breve{O}$ and *O* cannot be synchronized, albeit the two spatially far apart observers are physically stationary relative to one another. These conditions imply the spacetime geometry (or metrics) at *O* and $\breve{O}$ are different, a result of the fictitious oscillation’s effects on $\breve{O}$.

As previously described, observer $\breve{O}$ situated on the thin shell $\Sigma _0$ exhibits behavior that is in certain respects analogous to, yet fundamentally distinct from, that of an observer undergoing oscillatory motion in a spatial frame. In flat spacetime, an accelerating (or oscillating) observer is in a non-inertial reference frame. Within this frame, a fictitious (inertial) force arises due to the observer’s own acceleration. An inertial observer would appear to be accelerating (or oscillating) from the perspective of the non-inertial observer. In contrast, for the observer $\breve{O}$, only fictitious (inertial) observers appear to oscillate relative to the shell. Both $\breve{O}$ and inertial observers at spatial infinity remain physically stationary with respect to one another. Just as fictitious forces arise in non-inertial frames, fictitious oscillations emerge from the curved spacetime geometries induced by a proper-time oscillator.

The fictitious oscillations under consideration are geometric features of spacetime, involving no transport of matter and hence no violation of causality—even in the limit where their apparent instantaneous velocity approaches infinity. These oscillations are not physically realizable or directly observable; rather, they are mathematical artifacts arising from the Fourier decomposition of the proper-time oscillation. Although not measurable in a physical sense, their hypothetical influence on the temporal and spatial measurements performed by a stationary observer can lead to observable effects. As we shall demonstrate, these effects are encoded in the exterior spacetime geometry of the fictitious shell. In this sense, while fictitious oscillations are inherently unphysical, they can nonetheless yield measurable consequences within the spacetime framework experienced by an observer.

As we will discuss in “Conservation of intrinsic energy and restoring action” section, the total intrinsic mass-energy of a proper time oscillator is conserved. According to Noether’s theorem, a conserved energy system implies a time-translational symmetry. Under this symmetry condition, the fictitious radial oscillations’ total spacetime geometrical effects on $\breve{O}$ are constant over time. However, before we proceed further, we shall recall that the instantaneous velocity and displacement of the fictitious radial oscillation are both approaching infinity. To apply our knowledge in relativity, we will first study a thin shell with a finite radius and a subluminal instantaneous fictitious velocity.

### Thin shell with finite fictitious radial oscillations

In ref.^15^, we investigated a similar timelike hypersurface $\Sigma$ with finite radius $\breve{r}$. On the surface of $\Sigma$, we apply the same fictitious oscillations but with subluminal instantaneous velocities $\bar{v}_{f}({t})$, i.e.,

68 $$\begin{aligned} & \bar{t}_f(t,\breve{r})=t, \end{aligned}$$

69 $$\begin{aligned} & \bar{r}_f(t,\breve{r})=\breve{r}+\Re \cos (\omega _0 t), \end{aligned}$$

70 $$\begin{aligned} & \bar{v}_{f}({t},\breve{r})=\frac{\partial \bar{r}_{f}({t},\breve{r})}{\partial {t}}=-{\Re } \omega _0 \sin (\omega _0 {t}), \end{aligned}$$

where $\Re$ is the amplitude of radial oscillation and ${\Re } \omega _0<1$. The system is considered to have a time translational symmetry, the same condition for the thin shell $\Sigma _0$ with infinitesimal radius. We can apply relativity to analyze the effects on the observer $\breve{O}$ stationary on the thin shell’s surface. Under the time translational symmetry, the spacetime geometrical effects generated by the fictitious radial oscillations are constant over time.

In the present hypothetical construction, the spacetime exterior to the thin shell $\Sigma$ is a vacuum. An observer $\breve{O}$, comoving with the shell is non-inertial, effectively undergoing oscillatory motion relative to an inertial frame due to the influence of the fictitious oscillations. Crucially, these oscillations do not correspond to any physical transport of matter; rather, they represent purely geometric features of the spacetime within the context of the model. To elucidate their geometric consequences, we begin by analyzing their hypothetical impact on temporal and spatial measurements as observed by a stationary observer $\breve{O}$ located on the shell.

Observer $\breve{O}$ on the thin shell is stationary relative to observer *O* at spatial infinity. We can express the infinitesimal coordinate increments (*dt*, *dr*) of two events observed by *O* in terms of the infinitesimal coordinate increments $(d\breve{t},d\breve{r})$, for the same two events observed by $\breve{O}$,

71 $$\begin{aligned} \left[ \begin{array}{ c } dt \\ dr \end{array} \right] = \left[ \begin{array}{ c c } {\Upsilon ^t}_{\breve{t}} & 0 \\ 0 & {\Upsilon ^r}_{\breve{r}} \end{array} \right] \left[ \begin{array}{ c } d\breve{t} \\ d\breve{r} \end{array} \right] . \end{aligned}$$

The two off-diagonal terms of the transformation matrix $\Upsilon$ are zeros, which are deduced from the following: (1) The basis vectors of *O* and $\breve{O}$ are parallel for two observers stationary relative to one another, i.e., $\vec {e}_{\breve{t}} \parallel \vec {e}_{t}$ and $\vec {e}_{\breve{r}} \parallel \vec {e}_{r}$. 2) The basis vectors in the temporal and spatial directions are orthogonal in the local frames of *O* and $\breve{O}$, i,e., $\vec {e}_{t} \cdot \vec {e}_{r}=0$ and $\vec {e}_{\breve{t}} \cdot \vec {e}_{\breve{r}}=0$. Under the two conditions, we have

72 $$\begin{aligned} \Upsilon ^t_{\breve{r}}= & \vec {e}_{t} \cdot \vec {e}_{\breve{r}}=0, \end{aligned}$$

73 $$\begin{aligned} \Upsilon ^r_{\breve{t}}= & \vec {e}_{r} \cdot \vec {e}_{\breve{t}}=0. \end{aligned}$$

At $t=t_m=\pi /(2\omega _0)$, the fictitious displacement $\bar{r}_d$(=$\bar{r}_f-\breve{r}$) from Eq. (69) is zero, but the instantaneous velocity from Eq. (70) is,

74 $$\begin{aligned} \bar{v}_{f}(t_m,\breve{r})=\bar{v}_{fm}=-{\Re } \omega _0. \end{aligned}$$

Therefore, observer $\breve{O}$ on the thin shell is traveling at a velocity $\underline{v}_{fm} =-\bar{v}_{fm}(={\Re } \omega _0<1)$ relative to the fictitious inertial observer $\underline{O}$ without displacement from the equilibrium. We can apply relativity to study the properties of a moving observer, albeit the motion is in a fictitious frame. At this instant, the measurements by $\breve{O}$ will undergo length contraction and time dilation relative to the fictitious observer $\underline{O}$. However, as we shall recall, $\underline{O}$ is a fictitious inertial observer with its clock synchronized with *O* at spatial infinity. Although $\breve{O}$ remains stationary with *O*, its measurements will undergo the same length contraction and time dilation relative to *O*. Based on these arguments, we can write the two diagonal terms of the transformation matrix $\Upsilon$ as,

75 $$\begin{aligned} {\Upsilon ^t}_{\breve{t}}= & [1-(\bar{v}_{fm})^2]^{-1/2}=(1-{\Re }^2 \omega _0^2)^{-1/2}, \end{aligned}$$

76 $$\begin{aligned} {\Upsilon ^r}_{\breve{r}}= & [1-(\bar{v}_{fm})^2]^{1/2}=(1-{\Re }^2 \omega _0^2)^{1/2}. \end{aligned}$$

Since the system has a time translational symmetry, we can extend these results to all other times. Based on Eqs. (71), (75), (76), and the time translational symmetry, the line element on the thin shell $\Sigma$ is a constant over time, i.e.,

77 $$\begin{aligned} ds^2=[1-{\Re }^2 \omega _0^2]dt^2-[1-{\Re }^2 \omega _0^2]^{-1}dr^2-\breve{r}^2d\Omega ^2. \end{aligned}$$

As viewed from the exterior, the shell’s induced metric is,

78 $$\begin{aligned} ds^2_\Sigma =[1-{\Re }^2 \omega _0^2]dt^2-\breve{r}^2d\Omega ^2. \end{aligned}$$

Analysis for the line element on the surface of a thin shell with fictitious oscillations can be found in refs.^15,16^.

Under time translational symmetry, the fictitious oscillation’s effects are constant over time after taking into account the fictitious radial displacement,

79 $$\begin{aligned} \bar{r}_{fd}(t,\breve{r})=\bar{r}_f(t,\breve{r})-\breve{r}=\Re \cos (\omega _0 t). \end{aligned}$$

Together with the fictitious instantaneous velocity $\bar{v}_{f}({t},\breve{r})$ from Eq. (70), the line element from Eq. (77) can be written as,

80 $$\begin{aligned} ds^2=[1-(\omega _0^2\bar{r}_{fd}^2+\bar{v}_{f}^2)]dt^2-[1-(\omega _0^2\bar{r}_{fd}^2+\bar{v}_{f}^2)]^{-1}dr^2-\breve{r}^2d\Omega ^2. \end{aligned}$$

Although both $\bar{v}_{f}$ and $\bar{r}_{fd}$ vary over time, their combined effects are constant over time. This property is analogous to the constant total mass-energy of a particle generated from the displaced time $t_{d}$ and the displaced time rate $u_{d}$ as shown in Eq. (56).

### Schwarzschild spacetime

From Eq. (77), the line element on the surface of the thin shell $\Sigma$ is Schwarzschild-like. The fictitious oscillations localized on the shell’s exterior surface contribute to its gravitational influence, modifying the spacetime geometry. Although the spacetime remains asymptotically flat at spatial infinity, the induced geometry on the shell differs from this asymptotic structure, indicating that the intervening spacetime is curved.

The vacuum Einstein field equations, $R_{\mu \nu }=0$, govern the geometry of spacetime in the absence of matter. The unique, static, spherically symmetric, and asymptotically flat solution to these equations is the Schwarzschild metric. Given the metric at $r=\breve{r}$ from Eq. (77), the Schwarzschild solution that matches this boundary condition is:

81 $$\begin{aligned} ds^2 = \left( 1 - \frac{\breve{r} \mathcal {R}^2 \omega _0^2}{r} \right) dt^2 - \left( 1 - \frac{\breve{r} \mathcal {R}^2 \omega _0^2}{r} \right) ^{-1} dr^2 - r^2 d\Omega ^2. \end{aligned}$$

Note that our theory made no modifications to Einstein’s field equations.

The thin shell $\Sigma$ with fictitious oscillations is introduced as a hypothetical construct. The objective of this analysis is not to examine whether a physically realizable massive shell of finite radius can produce such oscillations, but rather to study the properties of this idealized system. Ultimately, the insights gained here will be extended to a limiting case: a fictitious shell of infinitesimal radius $\Sigma _0$ driven by a proper time oscillator. Importantly, while the oscillations themselves are modeled rather than derived from fundamental dynamics, we can nevertheless demonstrate that their gravitational influence on the exterior spacetime is equivalent to that produced by a massive thin shell.

A massive thin shell of radius $\breve{r}$ can be characterized by its effective mass *m*, surface energy density $\sigma$, and surface pressure *p*. To establish an equivalence between a massive thin shell and a fictitious oscillating shell, we define:

82 $$\begin{aligned} m= & \frac{\breve{r} \mathcal {R}^2 \omega _0^2}{2}, \end{aligned}$$

83 $$\begin{aligned} \sigma= & -\frac{1}{4\pi \breve{r}} \left( \sqrt{1 - \frac{2m}{\breve{r}}} - 1 \right) = -\frac{1}{4\pi \breve{r}} \left( \sqrt{1 - \mathcal {R}^2 \omega _0^2} - 1 \right) , \end{aligned}$$

84 $$\begin{aligned} p= & \frac{1}{8\pi \breve{r}} \left( \frac{1 - m/\breve{r}}{\sqrt{1 - 2m/\breve{r}}} - 1 \right) = \frac{1}{8\pi \breve{r}} \left( \frac{1 - \mathcal {R}^2 \omega _0^2 / 2}{\sqrt{1 - \mathcal {R}^2 \omega _0^2}} - 1 \right) . \end{aligned}$$

For a massive thin shell, these effective quantities are essential for establishing the correspondence between the shell’s properties and the gravitational field in the exterior spacetime. This relationship is rigorously defined by the Israel junction conditions, which relate the discontinuity in the extrinsic curvature across the massive shell’s surface to its surface stress-energy tensor.

The exterior Schwarzschild spacetime metric outside this massive thin shell is given by^21^:

85 $$\begin{aligned} ds^2=\left[ 1-\frac{2m}{r}\right] dt^2-\left[ 1-\frac{2m}{r}\right] ^{-1}dr^2-r^2d\Omega ^2=\left[ 1-\frac{\breve{r} {\Re }^2\omega _0^2}{r}\right] dt^2-\left[ 1-\frac{\breve{r} {\Re }^2\omega _0^2}{r}\right] ^{-1}dr^2-r^2 d\Omega ^2, \end{aligned}$$

which is precisely the same as the one previously derived from the fictitious oscillating shell (Eq. (81))—thereby establishing the equivalence of their external gravitational effects. Under this hypothetical premise, the fictitious oscillation amplitude corresponding to a massive thin shell with effective mass *m* is:

86 $$\begin{aligned} {\Re }=\sqrt{\frac{2m}{\breve{r}\omega _0^2}}. \end{aligned}$$

As purely spacetime geometrical effects, the fictitious oscillations determine the metric on the surface $\Sigma$, as well as the geometry of the surrounding spacetime. These gravitational effects uphold the principle of causality, as their propagation is constrained by the speed of light. Crucially, the fictitious oscillations do not induce any superluminal transfer of information.

By Birkhoff’s theorem^22,23^, a spherically symmetric vacuum solution must be static and asymptotically flat, and is uniquely given by the Schwarzschild metric. As a result, a massive thin shell can be contracted ($\breve{r} \rightarrow \breve{r}'$) while the external geometry remains Schwarzschild as long as the effective mass *m* remains constant. Given the hypothetical premise that the fictitious oscillations produce the same gravitational effects as a massive thin shell, the thin shell $\Sigma$ can likewise be contracted without altering the exterior field—as long as the equivalent mass *m*, defined by Eq. (82), remains constant. For a contracted shell of radius $\breve{r}'$, the corresponding fictitious oscillation amplitude $\Re '$ is determined from Eq. (86) as ${\Re }'=\sqrt{{2m}/({\breve{r}'\omega _0^2})}$.

As the thin shell $\Sigma$ is contracted to $\breve{r}=2m$, it encounters a coordinate singularity where the fictitious instantaneous velocity formally reaches the speed of light. Despite this, the amplitude $\Re$ determined from Eq. (86) and the related spacetime curvature tensors (e.g., the coordinate independent Kretschmann invariant^24^, $R^{\alpha \beta \gamma \delta }R_{\alpha \beta \gamma \delta }=48m^2/r^6= 12\breve{r}^2 \breve{\Re }^4\omega _0^4/r^6$, etc.) derived from the metric, remain well-defined as $\breve{r}\rightarrow 0$. Although the fictitious velocity formally exceeds the speed of light in this limit, there is no violation of causality or special relativity because these oscillations are purely geometric in nature and do not involve the transport of physical matter or information through spacetime.

According to general relativity, the structure of Schwarzschild spacetime undergoes a fundamental transformation beyond a black hole’s event horizon, where the roles of temporal and spatial coordinates effectively interchange. In the present case, our model inside the event horizon involves an instantaneous fictitious velocity exceeding the speed of light, rendering methods applicable to subluminal fictitious velocities inapplicable. Nevertheless, the extended metric given in Eq. (85) establishes a unique framework for understanding how such superluminal fictitious oscillations influence an observer’s measurements. Consequently, the model establishes a theoretical connection between the superluminal fictitious oscillations and the geometry of the exterior Schwarzschild spacetime.

The thin shell $\Sigma$ can be continuously contracted to an infinitesimal radius, with $\Re \rightarrow \infty$. This process yields a final configuration, which precisely matches the fictitious shell with an infinitesimal radius, $\Sigma _0$, generated by the proper time oscillator, as described in Eqs. (66) and (67). Our results confirm that the spacetime exterior to a stationary proper time oscillator is described by the Schwarzschild solution.

An idealized massive thin shell, when contracted to an infinitesimal scale, becomes equivalent to a point mass. The action and spacetime properties associated with both a massive thin shell and a point mass are well-defined within the framework of general relativity, which have been studied extensively in the literature. Importantly, our theory introduces no modifications to Einstein’s field equations; the exterior spacetime remains Schwarzschild.

For a proper time oscillator, its equivalence to a point mass implies that it inherits the same gravitational properties predicted by general relativity. In particular, its gravitational action is given by:

87 $$\begin{aligned} S = S_{\text {EH}} + S_{\text {GHY}} - S_{\text {flat}} = \frac{1}{16\pi } \int _{\mathcal {M}} d^4x \, \sqrt{-g} \, R + \frac{1}{8\pi } \int _{\partial \mathcal {M}} d^3x \, \sqrt{|h|} \left( K - K_0 \right) , \end{aligned}$$

where $S_{\text {EH}}$ is the Einstein-Hilbert bulk term, $S_{\text {GHY}}$ is the Gibbons-Hawking-York boundary term, and $S_{\text {flat}}$ is the subtraction term corresponding to flat spacetime.

For the Schwarzschild solution, the Ricci scalar vanishes ($R = 0$), and hence the Einstein-Hilbert term contributes nothing on-shell: $S_{\text {EH}} = 0$. Expressing the equivalent mass $m$ of the proper time oscillator in terms of its proper time amplitude $T_0$, via the de Broglie relation and Eq. (46), we write $m = \omega _0 = {1}/{T_0}$. The trace of the extrinsic curvature $K$ for a timelike hypersurface at constant radius $r$ in Schwarzschild spacetime is then given by:

88 $$\begin{aligned} K = \frac{2}{r} \left( 1 - \frac{2m}{r} \right) ^{1/2} + \frac{m}{r^2} \left( 1 - \frac{2m}{r} \right) ^{-1/2} = \frac{2}{r} \left( 1 - \frac{2}{T_0 r} \right) ^{1/2} + \frac{1}{T_0 r^2} \left( 1 - \frac{2}{T_0 r} \right) ^{-1/2}, \end{aligned}$$

where in the second expression we have substituted $m = 1/T_0$. Here, $K_0 =2/r$ denotes the trace of the extrinsic curvature of the boundary $\partial \mathcal {M}$ when embedded in flat spacetime.

The exterior spacetime outside a proper time oscillator is curved. Here, we show that matter with proper time oscillation can link directly with spacetime. The theory paints a simple picture: ‘The proper time oscillator exerts fictitious radial oscillations on a thin shell with an infinitesimal radius. These radial oscillations alter the spacetime metric on the thin shell’s surface and curve the surrounding external spacetime. In turn, the curved spacetime tells other matter how to react in the presence of the proper time oscillator’^16^.

The spacetime geometry in our model has a true singularity at $r=\epsilon /2$, where any causal timelike or null geodesic cannot be extended further. This result is not precisely the Schwarzschild metric since the singularity is not exactly at some non-zero $\epsilon$. However, there is no mystery about what spacetime structure is cloistered behind this singularity; the hidden geometry is our proper time oscillation. As shown, a classical proper time oscillator has the gravitational effects of a point mass in relativity except the singularities are on a thin shell with infinitesimal radius.

## Forward in time

The proper time oscillator observed in the quantized real scalar field, $\zeta (x)$, is the same oscillator that generates a Schwarzschild field when idealized as classical and stationary in space. The varying time rate is not the product of motion in space or a gravitational field. In this section, we examine the flow of time in a proper time oscillator.

Suppose the proper time oscillator carries an internal clock. The internal time read will reflect the fluctuating time of the underlying spacetime. Here, the concept of “internal time” is used within theoretical frameworks to describe the evolution of a quantum system, allowing for the study of time-dependent quantum phenomena. However, this internal clock is not a physical object within the particle but a mathematical construct used in analysis. The clock in the proper time oscillator is the internal clock hypothesized by de Broglie^20^. Although not conclusive, experimental data is found to be compatible with de Broglie’s conjecture^25^. In our theory, fluctuations in internal time can give rise to measurable variations in the decay rates of unstable particles. This effect will be discussed in “Magnitudes of the oscillations” section.

Consider the idealized classical proper time oscillator studied in Section “Gravitational field with proper time oscillation as a source”. The oscillator has an angular frequency of $\omega _0$ and amplitude $\mathring{T}_0=1/\omega _0$. Its internal time from Eq. (62) is,

$$\begin{aligned} \mathring{t}_{f}=t+\mathring{t}_{d}=t-\frac{\sin (\omega _0 t)}{\omega _0}. \end{aligned}$$

The internal time rate of the oscillator relative to the coordinate time is,

89 $$\begin{aligned} \mathring{R}_t=\frac{\partial \mathring{t}_{f}}{\partial t}=1-\cos (\omega _0t), \end{aligned}$$

which is bounded between 0 and 2. As a result, the sinuating internal time $\mathring{t}_f$ bounces along its geodesic but never goes back to its past. An oscillator’s internal time flows only forward. The average internal time rate is the same as the coordinate time. If the angular frequency is rapid, detecting its effects will be challenging. An oscillator will appear to propagate along a smooth time-like geodesic in an experiment if the instrument is not sensitive enough to pick up the oscillation.

**Fig. 1:**
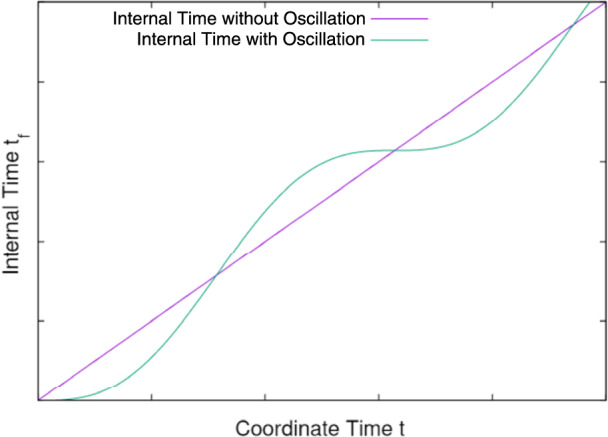
Proper time oscillation of a particle. The internal time never travels back to the past.

Figure 1 depicts the nature of the proper time oscillation. The purple line is the ’smooth’ internal time without oscillation. The green line is the internal time of a particle with oscillation obtained from Eq. (62). The cycle is repeated, but there is not a single moment that the internal time reverts to its past. An idealized classical proper time oscillator does not travel backward in time.

As a quantum system, there is a probability distribution of the proper time oscillator’s internal time. In Eq. (59), we propose an uncertainty relation between the temporal displacement $t_d$ and temporal momentum $P_t$. From Eqs. (5), (8) and (57), $t_d$ and $P_t$ are related to the internal time $t_f$ and internal time rate $R_t$, i.e.,

$$\begin{aligned} & {t}_{f}=t+{t}_{d}, \\ & R_t=1+\frac{P_t}{m}. \end{aligned}$$

Based on Eq. (59), $t_f$ and $R_t$ satisfy an uncertainty relation,

90 $$\begin{aligned} \Delta t_f\Delta R_t \ge \frac{1}{2m}. \end{aligned}$$

For a one-particle state, we can make an interpretation based on what we have learned from Heinsenberg’s uncertainty relation: The uncertainty for a quantum harmonic oscillator in time signifies that the more precisely we know the internal time within a cycle of oscillation, the less precisely we can know its internal time rate, and vice versa, reflecting the inherent uncertainty in quantum mechanics.

In “Excitation of spacetime” section, we demonstrate that a proper time oscillator in a quantized field can only oscillate with a frequency $\omega _0$ and an amplitude $\mathring{T}_0=1/\omega _0$. Therefore, the internal time of an oscillator can never be observed with a displacement more than $\mathring{T}_0$ from the equilibrium coordinate time. On the other hand, the internal time rate of an oscillator with the unique frequency $\omega _0$ and amplitude $\mathring{T}_0$ is bounded between 0 and 2, as discussed above. Therefore, the internal time and internal time rate are bounded by the limits,

91 $$\begin{aligned} & t-1/\omega _0 \le t_f \le t+1/\omega _0, \end{aligned}$$

92 $$\begin{aligned} & 0 \le R_t \le 2. \end{aligned}$$

Despite the oscillator’s quantum nature, the deviation of the internal time from the coordinate time is minimal, confined to a maximum displacement of $\mathring{T}_0$. With the internal time rate $R_t \ge 0$ at all times, the internal time of an oscillator can never travel back to its past. In “Magnitudes of the oscillations” section, we will examine the magnitude of the oscillation for real particles, assuming they are also proper time oscillators.

## Conservation of intrinsic energy and restoring action

In “Proper time oscillation” section, we have identified an analogous example of the proper time oscillator. The system contains a particle with spatial translational motion and oscillation. An external spring mechanism exerts the restoring force. The system’s kinetic energy arises from the particle’s motion and oscillation; its potential energy is stored in the external spring mechanism. Together, the total energy of the system is conserved. In this section, we look into a similar restoring mechanism for the proper time oscillator.

The equilibrium of the proper time oscillation is the flowing coordinate time. A proper time oscillator is stationary in space at its rest frame with no external spring mechanism. Resistance against oscillation by local spacetime can provide the required restoring mechanism. For instance, when time in the underlying spacetime is excited and displaced from the equilibrium coordinate time, the local spacetime will have resistance against the displacement and tends to restore the system to equilibrium. This restoration action is analogous to the resistance of an elastic material to compression.

The conservation of the proper time oscillator’s intrinsic mass-energy is demonstrated in Eq. (56) for the Hamiltonian of a quantized field $\zeta _0'$ with oscillations in proper time only, i.e.,

93 $$\begin{aligned} H_0=\frac{m\omega _0^2 {t_d}^2}{2} + \frac{{P_t}^2}{2m}. \end{aligned}$$

For a one-particle state, the excitation of the field is a particle-like oscillator with mass-energy *m*. On the Hamiltonian equation’s RHS, the terms are the oscillator’s ‘potential’ and ‘kinetic’ energy. The ‘kinetic’ component arises from the temporal oscillation of the spacetime excitation. The ’potential’ component is stored in the spacetime-restoring mechanism due to the temporal displacement from the equilibrium. Both components are intrinsic energy stored and generated at the local spacetime underlying the excitation. No external force fields or spring mechanisms are responsible for the restoring action. The total intrinsic energy is the mass-energy of the non-interacting particle-like oscillator, which is conserved.

“Thin shell with finite fictitious radial oscillations” section shows that oscillations in proper time give rise to fictitious radial oscillations. Both the resulting fictitious displacement and instantaneous velocity contribute to gravitational effects. Together, their influence on the surrounding spacetime remains constant over time. The conserved energy of a classical proper time oscillator reflects the system’s time-translational symmetry. The resulting Schwarzschild solution aligns precisely with expectations for a static, spherically symmetric system in general relativity.

To understand the properties of an oscillator’s restoring mechanism that has both quantum and gravitational properties, we require a theory of quantum gravity. Unfortunately, we still lack a complete and experimentally verified theory. Despite that, by adopting a spacetime-restoring mechanism, we can reconcile the properties of a quantum particle and Schwarzschild field from proper time oscillation. The theory developed is consistent with the standard theories as shown in “Proper time oscillator vs. standard theories” section.

## Temporal and spatial oscillations symmetry

General relativity treats time and space on an equal footing, which differs from how time is treated in quantum mechanics. Time enters quantum mechanics as a parameter and not as an operator, contrasting the position $\textbf{x}$ of a particle, which is the eigenvalue of an operator. Attempts to promote time to an operator in quantum theory have met many difficulties. Here, we clarify that the symmetry between time and space examined in this paper is for harmonic oscillations and not the coordinate time.

One of the reasons why time is not treated as a self-adjoint operator can be traced back to Pauli. According to Pauli^17,18^, a time operator and the Hamiltonian of a system should satisfy a commutation relation, $[H,t] =-i$. As a universal time operator, its spectrum should continuously span the entire real line. If the Hamiltonian *H* forms a conjugate pair with the time operator *t*, its spectrum shall also be continuous and unbounded. However, this conclusion would contradict our observation that the Hamiltonian *H* for a physical system is typically bounded from below. Based on these reasonings, time is generally not treated as an operator in the standard formulations of quantum theory.

Another issue is that a time operator is surprisingly complicated to develop. In a relativistic theory, we can promote the coordinate time *t* as an operator. In that case, a particle’s proper time $\tau$ can be taken as the time parameter, allowing us to define operators $X^\mu (\tau )$ with $X^0=t$. However, this line of thinking has encountered a problem. “The many times are the problem; any monotonic function of $\tau$ is just as good a candidate as $\tau$ itself for the proper time, and this infinite redundancy of description must be understood and acccounted for”^26,27^. In quantum field theory, another approach has been adopted to put time and space on an equal footing; the position is demoted from an operator to match the status of time as a label.

In this paper, we have considered a symmetry between time and space in a matter field. However, the symmetry we have investigated is for harmonic oscillations and not coordinate time. As a proper time oscillator, the fluctuating time $t_f$ oscillates about the coordinate time *t*, i.e.,

$$\begin{aligned} t_d=t_f-t. \end{aligned}$$

When considering the temporal displacement $t_d$, the coordinate time *t* is the equilibrium point of the oscillation. This temporal oscillation is an analogy of the oscillation in space.

In “Quantum harmonic oscillator in time” section, the temporal displacement $t_d$ is treated as a self-adjoint operator analogous to its spatial counterpart. Coordinate time is a parameter and not an operator, the same as what is adopted in quantum theory. Therefore, time *t* and Hamiltonian *H* do not form a conjugate pair. There is no commutation relation that triggers the Pauli’s theorem. Whether there is a more profound symmetry between space and time in quantum theory, where coordinate time can be treated as an operator, is beyond the scope of our investigations.

The introduction of the temporal displacement $t_d$ and temporal momentum $P_t$ operators also have no conflict with Pauli’s theorem. The commutation relation established in Eq. (58) does not involve energy. The temporal displacement and energy do not form a conjugate pair. Therefore, there is no restriction on the temporal displacement spectrum by the system’s Hamiltonian, which is bounded from below. The temporal displacement and temporal momentum can be treated as self-adjoint operators without contradicting Pauli’s theorem.

## Proper time oscillator vs. standard theories

**Fig. 2:**
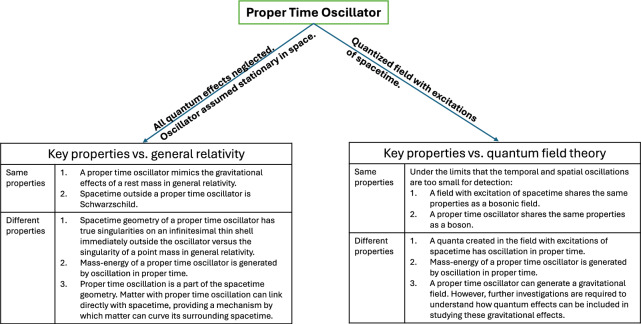
Schematic relationships between some of the key properties for proper time oscillators, general relativity and quantum field theory.

Let us review the properties of a proper time oscillator against quantum theory and general relativity. Regarding general relativity, we have demonstrated that the gravitational field of a proper time oscillator, when assumed stationary in space, is a Schwarzschild field, precisely as predicted by general relativity for a rest point mass. While our theory makes no modifications to Einstein’s field equations, the fictitious oscillation model has fundamental differences from the standard framework. Notably, gravitational singularities are located on a thin shell of infinitesimal radius, rather than at the center of rest mass as in conventional general relativity. Additional key distinctions are outlined in Fig. 2.

The model explains how the intrinsic structures of a proper time oscillator influence the spacetime geometry, establishing a direct correlation with spacetime. However, we should remember that the quantum effects were neglected when we developed the gravitational field. The proper time oscillator was assumed to be classical and stationary in the analysis. A complete quantum gravity theory is required to fully understand the gravitational properties of a proper time oscillator.

Quantum theory is one of the most quantitatively accurate theories in science. Numerous experiments have tested the theory, e.g., measurements of the electron magnetic moment^28^. As shown in “Plane wave with oscillations in time and space” section, a $\zeta$ field contains information for the oscillations of matter in the system. Applying Eqs. (18) and (19), the temporal and spatial oscillation displacements, $\zeta _t$ and $\zeta _\textbf{x}$, are the derivatives of $\zeta$ in respect to *t* and $\textbf{x}$ respectively. Also, as defined in Eq. (34), a real scalar field $\varphi$ can be derived from $\zeta$, where $\varphi$ satisfies the Klein-Gordon equation. Apart from the proper time oscillations, the real scalar field $\varphi$, derived from $\zeta$, has the exact properties of a bosonic field in quantum field theory.

Applying $\zeta$ to describe the properties of a real scalar field and its oscillations is analogous to using a four-potential $A^\mu$ in electromagnetic theory. The electric and magnetic fields, *E* and $\textbf{B}$, are components of the electromagnetic tensor, the exterior derivative of $A^\mu$. The properties of an electromagnetic field can be derived from the four-potential $A^\mu$ in lieu of explicitly in terms of the *E* and $\textbf{B}$ fields. Similarly, instead of explicitly using the temporal and spatial displacements, $\zeta _t$ and $\zeta _\textbf{x}$, it is sufficient to apply $\zeta$ in our formulations to describe the dynamics of a field with oscillations of matter in time and space. From $\zeta$, we can obtain $\zeta _t$ and $\zeta _\textbf{x}$.

The difference between the properties of a proper time oscillator and quantum theory is the additional oscillations in proper time. Suppose an experiment is not sensitive enough to detect the effects of the proper time oscillations. In that case, the properties of a proper time oscillator are the same as those of a quantum particle predicted in quantum theory until we reach an energy level, where the oscillations’ effects become significant. Figure 2 illustrates the schematic relationships between the key properties of a proper time oscillator, general relativity and quantum field theory.

## Magnitudes of the oscillations

Can a real particle also have oscillation in proper time? In other words, a real particle will be an excitation of the corresponding quantum field and its underlying spacetime. If it is true, the proper time oscillation will allow a real particle to acquire its mass-energy and interact directly with spacetime, generating a gravitational field. The properties of a real particle with proper time oscillation will be the same as predicted by quantum theory until its oscillation’s effects are significant enough for detection. Here, assuming real particles also oscillate in proper time, we examine the magnitude of their oscillations.

Firstly, consider the decay of particles. As shown in Eq. (2), the internal time of a particle evolves with oscillation, implying that the decay of an unstable particle has fluctuating rates. Let us examine the magnitude of the proper time oscillations for $\pi ^\pm$ ($\omega _0=2.1\times 10^{23 }s^{-1}$) and $\pi ^0$ ($\omega _0=2.0\times 10^{23 }s^{-1}$), which are the lightest spin-0 mesons. From Eq. (47), the amplitudes of the proper time oscillation for $\pi ^\pm$ and $\pi ^0$ are $4.8\times 10^{-24 }s$ and $5.0\times 10^{-24 }s$ respectively. Compared with the decay mean lifetime measured for the $\pi ^\pm$ of $(2.6033 \pm 0.0005)\times 10^{-8} s$ and $\pi ^0$ of $(8.5 \pm 0.2) \times 10^{-17} s$^29^, the proper time oscillations are small and beyond the resolutions that our experiments on particle decays can detect yet.

Next, consider a proper time oscillator in the quantized real scalar field $\zeta (x)$ with momentum $\textbf{k}$. From Eq. (50), the spacetime underlying the proper time oscillator has oscillations in time and space with an amplitude $\mathring{T}$ and $\mathring{\textbf{X}}$ respectively, i.e.,

94 $$\begin{aligned} \mathring{T}=\sqrt{\frac{\omega }{\omega _0^3}},\quad \mathring{\textbf{X}}=\frac{\textbf{k}}{\sqrt{\omega _0^3\omega }}. \end{aligned}$$

A particle-like proper time oscillator created at a coordinate $(t,\textbf{x})$ will be displaced to a coordinate $(t_f, \textbf{x}_f)$ due to the fluctuations of its underlying spacetime. Therefore, the created particle with momentum $\textbf{k}$ not only undergoes a spatial shift but also oscillates.

As we shall note in Eq. (94), the oscillations can be magnified by projecting the particle to a higher energy level, e.g., experiments on a particle’s arrival time. Let us examine the oscillation amplitudes for a $\pi ^+$. As shown in Table 1, the amplitudes are amplified when the particle’s speed is increased. At higher energy, the effects of the particle’s oscillations will be easier to detect. However, even for a $\pi ^+$ particle with an energy of 1 TeV, detecting the oscillations is still beyond the reach of our current experiments.

**Table 1:** Oscillation amplitudes of a $\pi ^+$ with different projected energies.

*E*(*GeV*)	$\mathring{T} (s)$	$\mathring{X} (m)$
1	$1.3\times 10^{-23}$	$3.5\times 10^{-15}$
10	$4.0\times 10^{-23}$	$1.2\times 10^{-14}$
100	$1.3\times 10^{-22}$	$3.8\times 10^{-14}$
1000	$4.0\times 10^{-22}$	$1.2\times 10^{-13}$

A heavier particle has smaller amplitudes than a lighter particle if both are projected to the same energy. Since $\pi ^+$ is already one of the lightest spin-0 massive bosons and its oscillations at 1 TeV are not yet detectable, the oscillations of all other massive bosons are also too small for detection. A real particle with proper time oscillation has the exact properties as predicted by quantum field theory until the effects of a particle’s oscillations become significant. However, those oscillations have not yet reached a level that are detectable.

So far, our discussions have been limited to bosons. What about fermions? If a boson’s mass-energy is generated by proper time oscillation, we expect the same can be true for a fermion. As an intrinsic property of a particle, the properties of mass are the same for all massive particles regardless of their spins. In the following discussions, we make an assumption that a spin-1/2 particle also has proper time oscillation. Whether this assumption is valid requires further examinations, which will be delayed to a future paper. Here, we aim to examine the magnitude of oscillations for fermions, assuming they also have proper time oscillations.

Let us consider an electron ($\omega _0=7.6\times 10^{20 }s^{-1}$), the lightest elementary particle apart from neutrinos. From Eq. (47), the amplitude of the proper time oscillation is $\mathring{T}_0=1.3\times 10^{-21 }s$. Projected at an energy of 1 TeV, the amplitudes from Eq. (94) are $\mathring{T}=1.8\times 10^{-18 }s$ and $\mathring{\textbf{X}}=5.6\times 10^{-10 }m$. Again, the oscillations are small for detection.

Next, consider a neutrino. As of today, the masses of the three neutrinos are unknown. However, active research has been carried out in this area, with the expectation that new physics could be revealed. Because of their extremely lightweight, neutrinos have much larger temporal and spatial oscillations than other particles.

To demonstrate the magnitude of the oscillations, we will assume a neutrino mass of $m=2eV$^30,31^. As shown in Table 2, the amplitudes are much larger than those for an electron with the same energy. Despite a neutrino is hard to detect, and its mass is unknown, the spatial amplitudes projected at high energy could be in the macroscopic scale, e.g., $\mathring{\textbf{X}}=7.0$ cm at $E=$1 Tev. Because they are extremely lightweight, neutrinos can be projected at very high speed, amplifying the oscillations for possible measurements.

**Table 2:** Oscillation amplitudes of a neutrino with different projected energies with assumed mass $m=2eV$.

*E*(*GeV*)	$\mathring{T} (s)$	$\mathring{X} (cm)$
1	$7.4\times 10^{-12}$	0.22
10	$2.3\times 10^{-11}$	0.70
100	$7.4\times 10^{-11}$	2.20
1000	$2.3\times 10^{-10}$	7.00

A neutrino possesses mass and therefore must travel at subluminal speeds, in accordance with special relativity. Numerous experiments have been conducted to measure the speed of neutrinos^32–35^, all of which have found no deviations from the expected behavior within experimental uncertainty. Nonetheless, continued measurements are motivated by speculative theoretical models that predict potential deviations under certain conditions, such as tachyonic neutrinos or Lorentz-violating oscillations. In the context of quantum gravity, it has been proposed that particles propagating through a fluctuating spacetime—subject to so-called lightcone fluctuations^36–38^—may experience quantum-induced uncertainties in travel time. Over cosmological distances, these fluctuations could accumulate, leading to a measurable spread in arrival times. This uncertainty is often modeled by a power-law of the form $\Delta t' \propto l^mE^n$, where *l* is the distance traveled, *E* is the energy of the particle, and *m*, *n* are model-dependent parameters^39–41^. Although no such velocity fluctuations have been observed so far, it has been suggested that quantum gravitational effects might become observable for neutrinos with energies exceeding the *TeV* scale^40^. However, the arrival time uncertainty for neutrinos from astrophysical and cosmological sources depends heavily on the source type, the detection method, and the neutrino energy. Observations to date are not conclusive, primarily due to limited event statistics, uncertainties in the emission time of neutrinos relative to photons or gravitational waves, and the challenges of associating detected neutrinos with specific astrophysical events.

As established in “Excitation of spacetime” section, a particle characterized by momentum $\textbf{k}$ exhibits an oscillation possessing a quantized spatial amplitude, $\mathring{\textbf{X}}={\textbf{k}}/{\sqrt{\omega _0^3\omega }}$. Due to the quantum mechanical nature of this phenomenon, the observation of a particle at different phases of its oscillation cycle introduces an intrinsic uncertainty in its observed spatial location, expressed as:

95 $$\begin{aligned} \Delta \textbf{x}'=\frac{\textbf{k}}{\sqrt{2\omega _0^3\omega }}. \end{aligned}$$

Extending this to a collective scenario, consider a large ensemble of particles possessing identical momentum $\textbf{k}$ (and consequently, an average velocity $\textbf{v}_{avg}=\textbf{k}/\omega$) that are emitted simultaneously from a common source. The aforementioned spatial oscillations fundamentally contribute to an uncertainty in their arrival time at a designated target, which is quantified by:

96 $$\begin{aligned} \Delta t'=\frac{\Delta \textbf{x}'}{\textbf{v}_{avg}}=\sqrt{\frac{\omega }{2\omega _0^3}}\propto E^{1/2}. \end{aligned}$$

**Fig. 3:**
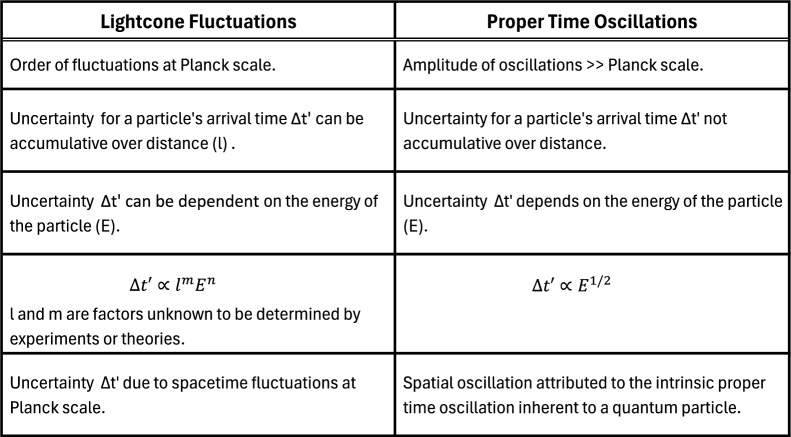
Several key divergences between the arrival time uncertainty associated with lightcone fluctuations and proper time oscillations.

The result derived from Eq. (96) bears a strong resemblance to the power-law behavior frequently considered within the realm of quantum gravity. Nevertheless, this observed effect is attributed to an intrinsic proper time oscillation inherent to a quantum particle. Crucially, this phenomenon can manifest over distances considerably shorter than the cosmological scales at which spacetime fluctuations are typically investigated. As we shall also note, the uncertainty exhibits a dependence on the particle’s mass, a parameter yet to be precisely determined for neutrinos. Should our assumption regarding the proper time oscillation of neutrinos prove valid, experimental investigations involving neutrinos could furnish critical insights into verifying whether a particle can indeed exhibit such proper time oscillations. Figure 3 elucidates several key divergences between the arrival time uncertainty associated with lightcone fluctuations and that arising from proper time oscillations.

Spatial fluctuations arising from proper time oscillations could, in principle, become detectable at laboratory scales. Table 3 compares the neutrino time-of-flight measurements from the MINOS and OPERA experiments. As an illustrative example, a neutrino spatial oscillation amplitude of $|\textbf{X}| = 7.0$ cm at $E=1$ TeV (see Table 2) corresponds to an arrival time uncertainty of $1.6\times 10^{-10}$s from Eq. (96). The current experimental time resolution—on the order of nanoseconds ($10^{-9}$s)—is within one order of magnitude of this threshold, suggesting that detection may not be entirely out of reach. However, it is crucial to ensure that the experimental time resolution is significantly better than the characteristic timescale of the spatial oscillation; otherwise, the signal may be obscured by measurement uncertainty.

**Table 3:** Comparison of MINOS and OPERA neutrino time-of-flight measurements.^33,34^.

	OPERA	MINOS
Baseline distance (km)	$\sim 731$	$\sim 734$
Timing resolution (ns)	Final $\sim 1$, initial $\sim 60$	$\sim 2$–3 (systematic), $<1$ (statistical)
Speed deviation sensitivity $\left( \frac{v - c}{c}\right)$	$\lesssim 2 \times 10^{-6}$ (final)	$(1.0 \pm 1.1) \times 10^{-6}$
Typical neutrino energy (GeV)	Mean $\sim 17$ (range $\sim 1$–20)	Mean $\sim 3$ (extends up to $\sim 50$)

Existing accelerator-based neutrino beam experiments are not yet capable of routinely producing neutrinos with energies exceeding 1 TeV. To date, only rare events with neutrino energies approaching 100 GeV have been produced, while typical beam energies lie in the range of a few to several tens of GeV. Although theoretical predictions suggest an intriguing possibility for future detection, achieving direct experimental verification will require significant advances in both timing resolution and high-energy neutrino production.

In quantum gravity, the spacetime metric itself is subject to quantum fluctuations, as envisioned in spacetime foam models. Lightcone fluctuations in quantum gravity can challenge the classical causal structure and potentially lead to effective causality violations at the quantum level, though macroscopic causality may still be preserved. Since the lightcone is defined by the metric, quantum fluctuations in the metric imply an uncertainty in the causal structure—making it probabilistic or emergent rather than absolute. This can lead to situations where the classical temporal ordering of events becomes indefinite or observer-dependent at small scales. Understanding the quantum nature of spacetime and its implications for causality is one of the most conceptually profound and technically challenging aspects in the quest for a complete theory of quantum gravity.

In our theory, the arrival time of a particle carries an intrinsic uncertainty due to its spatial oscillations. If this idea is extended to photons, the average speed of light remains constant, but a quantum-level uncertainty in the photon’s propagation speed may arise. Similar to the framework of lightcone fluctuations, such uncertainty could potentially lead to effective violations of causality at microscopic scales. However, since photons have no proper time, it remains an open question whether they undergo spatial oscillations during propagation. Developing a theory that accounts for both temporal and spatial oscillations of massless particles is essential. Without such a theoretical framework, the question of causality violation in our theory cannot be addressed.

## Conclusions and discussions

As shown in this paper, a field that can excite its underlying spacetime has the basic properties of a quantum field^13,14^. A proper time oscillator has properties (e.g., commutation relation, uncertainty relation, etc.) resembling a quantum harmonic oscillator, except the oscillation is in time, not space. If a quantum harmonic oscillator has a counterpart in time, our results demonstrate that such an oscillator in time will have the properties of a quantum particle.

As a part of the spacetime geometry, a proper time oscillator can curve its surrounding spacetime and generate a gravitational field. Assuming the proper time oscillator is a classical object that remains stationary in space, the spacetime outside is a Schwarzschild field^15,16^. Therefore, if nature allows oscillation in time, a proper time oscillator can act as a gravitational source, allowing matter to interact directly with spacetime. However, quantum effects are neglected when we study the proper time oscillator’s gravitational field. Whether spacetime is quantized is also an unanswered question. Further investigations are required to understand how quantum effects can be included in studying a proper time oscillator’s gravitational effects.

Apart from investigating the properties of a proper time oscillator, we have discussed what physical systems can potentially cause their underlying spacetime to fluctuate. One of the examples is a black hole. As discussed, a classical proper time oscillator has the gravitational effects of a point mass in relativity. Theoretically, spacetime is curved if a black hole causes its underlying time to fluctuate. However, the nature inside a black hole cannot be directly revealed because of the strong gravitational effects. Understanding the true nature inside a black hole requires better knowledge of the quantum gravity theory. As we have demonstrated, a proper time oscillator has both quantum and gravitational properties. Whether these properties help us understand what happens inside a black hole may be worth exploring further.

A quantum field is essentially described as a mathematical sum of creation and annihilation operators with properties akin to those developed for a quantum harmonic oscillator. Although the natures of the two oscillators are different, their mathematical structures and operations share many common features. The two oscillators have some similar characteristics. (As we shall recall, the spatial position is demoted to a parameter in quantum field theory, allowing time and space to be treated on an equal footing. The position operator used for a quantum harmonic oscillator is not directly applicable in the framework of a quantum field. Despite the differences, if we look at how the operators for creation and annihilation operate for the two oscillators, they function similarly).

As discussed, in the context of time and space symmetry, if a system demonstrates specific characteristics in space, there might be systems with similar characteristics in time. The similarities in the mathematical structures between a bosonic field and a quantum harmonic oscillator could hint that a quantum particle has something to do with an oscillator in time.

In the last section, we have examined the possibility that the spacetime underlying a real particle also oscillates in time. If that is the case, the proper time oscillation will allow a real particle to interact directly with spacetime and generate a gravitational field. The properties of a real particle with proper time oscillation will be the same as predicted by quantum theory until the oscillation effects are significant enough for detection. Assuming real particles are also proper time oscillators, but with other intrinsic quantum properties, we have examined the magnitude of their oscillations.

Examination of the magnitude of oscillations for all known real particles reveals that they have yet to reach a level detectable by the current experiments. On the other hand, a real particle’s possible temporal and spatial oscillations are measurable physical quantities. Although their magnitudes are still too small to detect, their effects can be magnified by projecting a particle to higher energy, e.g., measuring the arrival time of a high-energy particle. As demonstrated, the magnitudes of these possible oscillations are much larger than the Planck scale. In particular, if a neutrino also has spacetime temporal and spatial oscillations, its magnitudes could be macroscopic when projected to very high energy, providing a better chance to be detected in future experiments. An analysis of the timing resolution and sensitivity of current experiments suggests that the spatial oscillation of a 1 TeV neutrino may not be entirely beyond the reach of observation. However, direct verification of such oscillations would require substantial advancements in both timing resolution and the production of high-energy neutrinos. If those oscillations are detected, the results could provide potential evidence of a proper time oscillator.

## Data Availability

All data generated or analyzed during this study are included in this published article.
